# Enhancement of growth media for extreme iron limitation in Escherichia coli

**DOI:** 10.1099/acmi.0.000735.v4

**Published:** 2024-06-03

**Authors:** James W. Southwell, Keith S. Wilson, Gavin H. Thomas, Anne-Kathrin Duhme-Klair

**Affiliations:** 1 Department of Chemistry, University of York, Heslington, York, YO10 5DD, UK; 2 York Structural Biology Laboratory, University of York, Heslington, York, YO10 5DD, UK; 3 Department of Biology, University of York, Wentworth Way, York, YO10 5DD, UK

**Keywords:** *Escherichia coli*, iron, siderophores

## Abstract

Iron is an essential nutrient for microbial growth and bacteria have evolved numerous routes to solubilize and scavenge this biometal, which is often present at very low concentrations in host tissue. We recently used a MOPS-based medium to induce iron limitation in *Escherichia coli* K-12 during the characterization of novel siderophore-conjugated antibiotics. In this study we confirm that growth media derived from commercially available M9 salts are unsuitable for studies of iron-limited growth, probably through the contamination of the sodium phosphate buffer components with over 100 µM iron. In contrast, MOPS-based media that are treated with metal-binding Chelex resin allow the free iron concentration to be reduced to growth-limiting levels. Despite these measures a small amount of *E. coli* growth is still observed in these iron-depleted media. By growing * E. coli* in conditions that theoretically increase the demand for iron-dependent enzymes, namely by replacing the glucose carbon source for acetate and by switching to a microaerobic atmosphere, we can reduce background growth even further. Finally, we demonstrate that by adding an exogeneous siderophore to the growth media which is poorly used by *E. coli*, we can completely prevent growth, perhaps mimicking the situation in host tissue. In conclusion, this short study provides practical experimental insight into low iron media and how to augment the growth conditions of *E. coli* for extreme iron-limited growth.

**Impact Statement**

Inside host organisms, free iron is an essential but scarce resource to bacteria due to high levels of competition with other microbes and the host organism itself. Thus, to impose biologically relevant conditions for the growth of bacteria in the laboratory, the media used must impose such conditions. To this end, we describe the optimization process that led to our previously reported zero-iron medium for the growth of *Escherichia coli* and evaluate the effect of microaerobic growth (2 % O_2_) and the addition of iron chelators, including the siderophore desferrioxamine. We advise zero-iron media, such as the one investigated in this work, be used in the development of antibacterials, especially in cases where bacterial iron uptake pathways are exploited for molecule internalization.

## Data Summary

The authors confirm all supporting data, code and protocols have been provided within the article. The composition of the zero-iron media described in this work was used by Thomas J. Sanderson, Conor M. Black, James W. Southwell, Ellis J. Wilde, Apurva Pandey, Reyme Herman, Gavin H. Thomas, Eszter Boros, Anne-Kathrin Duhme-Klair and Anne Routledge, *ACS Infect. Dis*. 2020, 6, 9, 2532–2541, https://doi.org/10.1021/acsinfecdis.0c00568.

## Introduction

Iron is an essential micronutrient for most living organisms and in aqueous media is most commonly found in its Fe(III) (ferric) oxidation state, which shows very low water solubility near neutral pH. To aid in the capture of the scarce Fe(III) cations many bacteria produce siderophores, low-molecular-weight compounds that possess a high affinity and selectivity for Fe(III) [1,3]. These compounds are biosynthesized and secreted by bacteria, fungi and some plants into their surrounding medium, to bind to and solubilize Fe(III), thereby facilitating its cellular uptake. Gram-negative bacteria, such as *Escherichia coli*, are able to sense their intracellular iron concentration using a repressor protein called Fur [3]. This protein acts at a transcriptional level and, when bound to intracellular Fe(II), binds to specific DNA regulatory sequences, to prevent their transcription [4,5]. The Fur regulon includes genes that are responsible for the biosynthesis of siderophores and their translocation into bacteria (e.g. cell-surface receptors) [6]. When bacteria experience iron-limiting conditions, intracellular levels of Fe(II) are depleted, such that remaining Fe(II) dissociates from Fur, depressing the expression of iron-regulated genes, including siderophore biosynthesis and uptake pathways for their corresponding iron complexes. Fur is a master iron regulator not only in Gram-negative but also Gram-positive pathogens such as *Staphylococcus aureus*. Whilst it is not well understood in all bacteria, and indeed some Gram-positive bacteria use other proteins (e.g. DrxR), its importance does have wider implications beyond *E. coli*. It is understood, however, that the major siderophore for *E. coli* is enterobactin (and its hydrolysis products), although citrate and desferrichrome are also used [3,7]. For comparison, *Campylobacter jejuni* use the hydrolysis products of enterobactin, but do not themselves produce the siderophores [8]. In this instance, these siderophores are referred to as xenosiderophores.

To understand siderophore function and to assess the potency of antibacterial siderophore conjugates, often called ‘Trojan Horse’ antibacterials, biological assays measuring activity against bacteria are required and various growth-based methods have been used for this purpose [9], with several examples reported in the literature [10,19]. Key to these methods is their ability to easily mimic the iron-limiting conditions found in the host organism while providing other nutrients required for rapid growth. This is usually achieved using one of two alternative approaches, namely rich media with iron-chelating agents added, or defined minimal medium with no added iron. The former method, which is easier to set up, uses undefined media such as lysogeny broth (LB) [20,21], tryptic soy broth (TSB) [22,23] and Müller-Hinton broth II (MHII) [12,18, 20, 21, 24], the last being the most popular choice for bacteria, including *E. coli*. Even though iron is not specifically added to these media, the iron levels are often too high to limit bacterial growth, and so additional measures are taken, such as the acid treatment of glassware, the avoidance of metal syringes (especially with acids) and Chelex treatment (an indiscriminate metal-chelating resin) [25,26]. It is also common for synthetic chelators, such as 2,2′-bipyridine (bpy), to be added to the media during bacterial growth assays to sequester iron, thereby reducing iron availability [27]. The overall formation constant (log β) represents the equilibrium between complex formation and corresponding free ligand(s) in solution. Higher values are therefore indicative of enhanced tendencies to form the corresponding complex. The log β for [Fe(bpy)_3_]^3+^ is 16.3, whereas those of the Fe(III) complexes of principal hexadentate siderophores, such as enterobactin and desferrioxamine B (DFO), are far higher, with values of 49.0 and 30.5, respectively. Hence these chelator treatments will not reduce free iron to levels where siderophore function becomes essential [2,3]. There are several literature examples of chelator addition to media in attempts to impose iron-limited conditions on bacteria but unless these chelators possess high iron selectivity, they can result in phenotypes that could be the result of depletion of multiple metals [28].

The latter strategy of ‘low iron media’ utilizes defined minimal media such as M9 media [7,21] and Tris-minimal succinate (TMS) media [29,31] for *E. coli* and *S. aureus*, respectively, that undergo similar treatments for iron removal. Since these media are made up of individual chemical components that can be Chelex treated individually, their iron levels are more easily controlled. In a recent study from our laboratory we developed a zero-iron medium for the assessment of Trojan Horse antibacterials against *E. coli* [32]. Here we describe the stages in the design of this medium, providing insight into process parameters and investigate methods to impose greater iron limitation, such as growth under microaerobic conditions and the addition of exogenous siderophores.

## Methods

### Media preparations

#### Materials

All materials were obtained from commercial suppliers such as Sigma Aldrich, Fisher Scientific, Fisher Bioreagents and VWR. Specifically, FeCl_3_.3H_2_O, Na_2_PO_4_, NaOH, NaOAc, desferrioxamine, 2,3-dihydroxybenzoic acid (DHB), the M9, minimal salts, 5× (M6030), and the Chelex 100 sodium form, 50–100 mesh (dry) resin (MFCD00163980) were purchased from Sigma Aldrich. The metal-free concentrated HCl was purchased from Fisher Scientific and the glucose, MgSO_4_ and CaCl_2_ from Fisher Bioreagents. The NH_4_Cl was purchased from VWR.

#### General remarks

All media were prepared from individual stock solutions and used in either sterile plastics (e.g. falcon tubes) or acid-treated glassware, avoiding the use of metal syringes. Each stock was made using Milli-Q pure water and stirred, in 10 % (w/v) Chelex 100 sodium form, 50–100 mesh (dry) resin (Sigma Aldrich), for 1 h and then filter sterilized before combining to give the desired media (except calcium and magnesium stocks, which were simply made up in chelexed Milli-Q water). Acid treatment involved the stirring of 6 M aqueous hydrochoric acid in the glassware for 18 h, followed by decanting, rinsing with acetone and oven drying.

#### Minimal M9 media

The following recipe was previously reported by Sanderson *et al*. [32]. To make a 10 ml stock, the following were combined: 7.779 ml Milli-Q pure water, 2 ml M9, minimal salts, 5× (Sigma Aldrich, M6030), 200 µl 20 % (w/v) glucose, 20 µl 1 M MgSO_4_ and 1 µl 1 M CaCl_2_. The final concentrations of each component in M9 media are as follows: 48 mM Na_2_HPO4, 22 mM KH_2_PO_4_, 19 mM NH_4_Cl, 9 mM NaCl, 22 mM glucose, 2 mM MgSO_4_ and 0.1 mM CaCl_2_. To make the acetate variation of this medium, the glucose component was simply replaced with 732 µl 1.67 mM NaOAc where less Milli-Q pure water is added (7.047 ml).

#### Zero-iron media

The following recipe is based on media reported by Neidhart *et al*. [33] and was previously reported by Sanderson *et al*. [32]. To make a 10 ml stock, the following were combined: 7.047 ml Milli-Q pure water, 1 ml 10× MOPS/Tricine stocks (400 and 40 mM, respectively), 1 ml 500 mM NaCl, 732 µl 1.67 mM NaOAc, 100 µl 132 mM K_2_HPO_4_, 100 µl 952 mM NH_4_Cl, 20 µl 1 M MgSO_4_ and 1 µl 1 M CaCl_2_. The resulting solution was then adjusted to pH 7.4, using chelexed 4 M NaOH. The final concentrations of each component in MOPS acetate are as follows: 40 mM MOPS, 4 mM Tricine, 50 mM NaCl, 0.12 mM NaOAc, 1.32 mM K_2_HPO_4_, 9.52 mM NH_4_Cl, 2 mM MgSO_4_ and 0.1 mM CaCl_2_. To make the glucose variation of this medium (not referred to as zero-iron medium), the acetate component was simply replaced with 200 µl 20 % (w/v) glucose where more Milli-Q pure water is added (7.779 ml).

#### Iron and siderophore stocks

In chelexed Milli-Q water, 1 M FeCl_3_ solutions were prepared from FeCl_3_.6H_2_O and then diluted into media stocks at 2.5 mM. These stocks were used for plate preparation. Siderophore stocks were made up similarly in chelexed Milli-Q water.

### Bacteria preparations

#### Bacterial strains

*E. coli* K-12 (BW25113) is a widely used K-12 derivative laboratory strain and was used throughout this study.

#### Bacteria stocks

For all bacterial growth assays, a bacterial stock was used which was prepared using the following procedure, previously reported by Sanderson *et al*. [32]. *E. coli* K-12 (BW25113) were grown overnight in M9 media with 10 % (w/v) casamino acids at 37°C and 180 r.p.m. Each sample was centrifuged at 3000 ***g*** and 4 °C and the solution decanted. The remaining solid was gently re-suspended in M9 media (10 ml), and centrifuged again, at 3000 ***g*** and 4 °C and the solution decanted (twice). The solid was then made to an OD_600_ of 0.05 in M9 media (20 ml) and grown for 24 h at 37 °C and 180 r.p.m. This overnight culture was then diluted by adding 0.4 ml to 0.1 ml chelexed glycerol in water and stored at −80 °C.

### Bacterial growth assays

#### Overnight culture preparation

Prior to all growth assay experiments, *E. coli* from the bacteria stock were grown for 22 h at 37 °C in M9 media supplemented with 10 % (w/v) casamino acids (total volume 20 ml), in an acid-treated conical flask, with shaking at 180 r.p.m. Optical densities from starter cultures were measured by dilution of 50 µl of starter culture in 950 µl of blank media, recorded to three decimal places using a Jenway 6305 spectrophotometer, or the cuvette mode of an Epoch 2 Microplate Spectrophotometer (Biotek), then multiplied to give the OD_600_ of the starter culture. This was carried out in plastic cuvettes with a 1 cm path length and are accurate to ±0.0005.

#### Plate preparation

Unless stated otherwise, growth assays were carried out once, in technical triplicates, using plate wells, with a starting OD_600_ of 0.05 (for initial media optimization studies) and 0.01 (for all subsequent experiments). Plate experiments used NUNC 96-well plates, with two-well deep edge wells filled with 200 µl water and full surrounding moats (2 ml). The only solvent added was water (no organic solvents).

#### Plate incubation procedure

Aerobically grown plates were incubated in an Epoch 2 Microplate Spectrophotometer (Biotek) at 37 °C, and shaken continuously in a double orbital pattern (282 r.p.m.). Absorbances at OD_600_ (OD_800_ for DHB siderophore studies, since their iron complexes absorb below this wavelength) for each well were recorded at *t*=0 and every 30 min, for 24 h. Microaerobically grown plates were incubated in a FLUOstar Omega microplate reader from BMG LABTECH at 37 °C, and shaken continuously in a double orbital pattern (180 r.p.m.) with attached Atmosphere Control Unit (ACU) ensuring a 2 % O_2_ atmosphere using an attached N_2_ gas cylinder. Absorbances at OD_800_ for each well were recorded at *t*=0 and every 30 min, for 75 h.

#### Data processing

Data were processed in Microsoft Excel software by first subtracting the average of the no-cell controls from all read data. All data were obtained in at least technical triplicate. For each cell condition, a mean average and error as standard deviation was calculated. Data were plotted using Origin software, as a growth curve. The mean average absorbance and its corresponding error for each well condition was taken at each time point. These data are shown as absorbance as a function of time.

## Results and discussion

### Optimizing a minimal medium for iron-limited *E. coli* growth

**Fig. 1.**
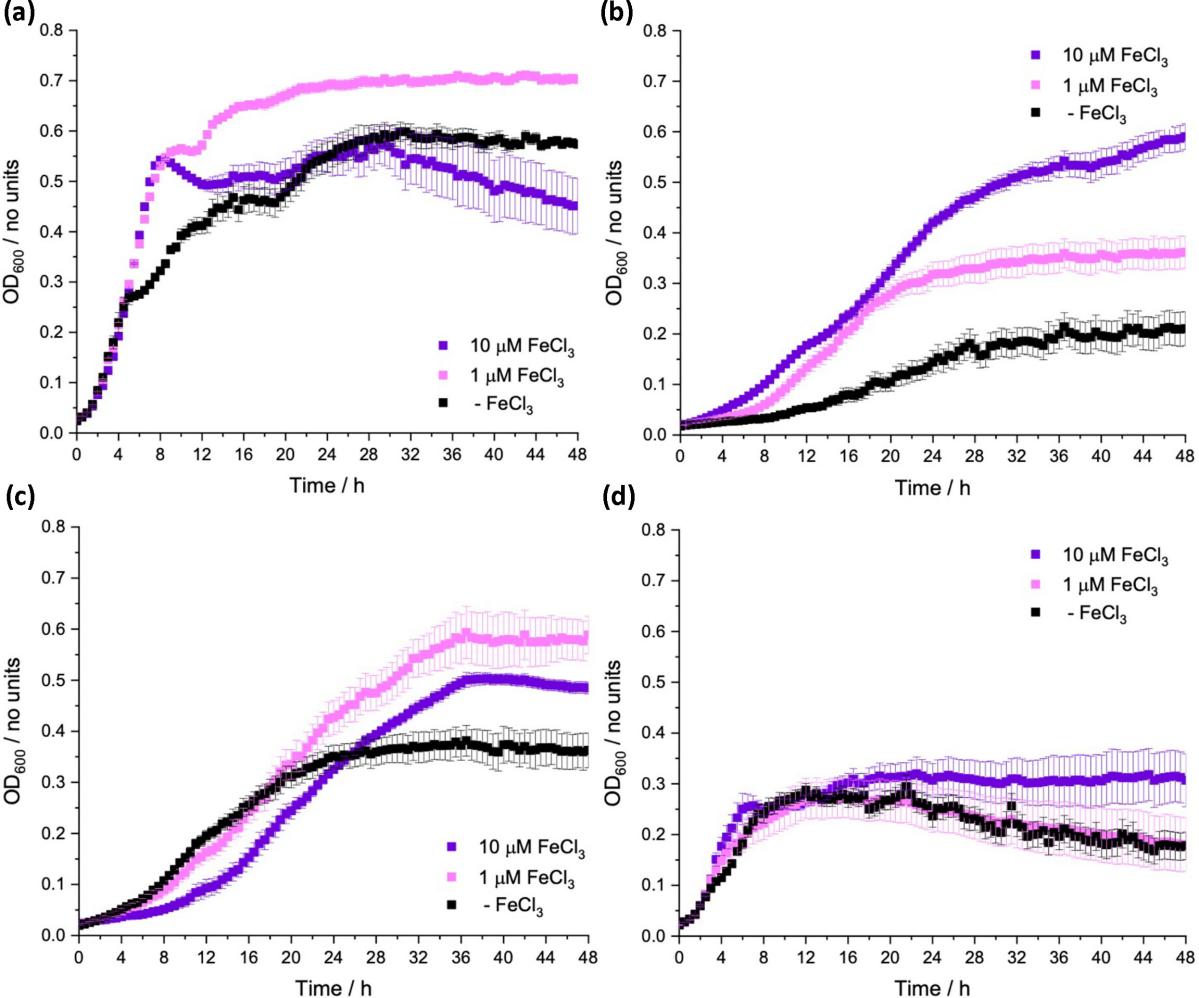
Growth of *E. coli* K-12 BW25113, over 48 h at 37 °C, with varying concentrations of supplemented FeCl_3_ in (**a**) M9 media with a glucose carbon source, (**b**) MOPS media with a glucose carbon source, (**c**) M9 media with an acetate carbon source and (**d**) MOPS media with an acetate carbon source (zero-iron media).

To study the function of *E. coli* in a defined minimal medium we initially tested the commonly used M9 medium, made using a 5× M9, minimal salts stock from Sigma Aldrich. However, despite the preparation of this medium including Chelex treatment (10 %, w/v), initial growth with no added Fe(III) was almost the same as that with either 1 or 10 µM of added FeCl_3_, demonstrating that this base medium is not appropriate for producing a consistent iron-limiting medium (Fig. 1a). A recent study by Soma *et al*. [34] suggests that the amount of iron that is present as a contaminant of the sodium phosphate component of M9 can vary enormously depending on its chemical purity and that this, in turn, correlates strongly with the growth rate of the bacterium [34]. In Neidhardt’s (1974) landmark paper on enterobacterial growth media, a MOPS-buffered medium was described where switching from a phosphate buffer component, with glucose as the carbon source, demonstrated a much lower background growth of *E. coli* in the absence of added iron [33]. Indeed we observed the phenotype indicative of a more iron-limited environment when the buffer was changed to MOPS (Fig. 1b*),* as well as the decreased background growth with no added iron observed by Neidhardt when switching the carbon source from glucose to acetate (Fig. 1c). Growth on acetate was proposed by Neidhardt to require more iron proteins for its metabolism [33], exemplified by aconitase B (AcnB), an essential enzyme required for growth on acetate that contains a [4Fe–4S] cluster [35].

Our final composition for the medium published by Sanderson *et al*. [32] is a hybrid of the media reported by Neidhardt that differs by Chelex treatment of the individual stock components, which is followed by re-introduction of the other metal salts present in M9, as Chelex is an indiscriminate metal binder. This medium, used in Fig. 1(d), was found to contain free iron below the detection limit by inductively coupled plasma MS and will thus be referred to as the zero-iron media [32]. In striking contrast, measurement from the M9-based media indicated a high amount of iron, 147 µM (8.2 mg l^−1^).

### Microaerobic growth of *E. coli* is impacted more by iron limitation

Given the suggestion from our earlier experiments that acetate increases the stringency of iron limitation compared to glucose and that this might be due to increased demand for iron-containing enzymes, we wished to test another growth state of *E. coli* which might similarly require increased iron–protein use. We therefore investigated microaerobic growth, a physiological state that *E. coli* encounters in the mammalian gut where siderophore function is important [36,37].

**Fig. 2.**
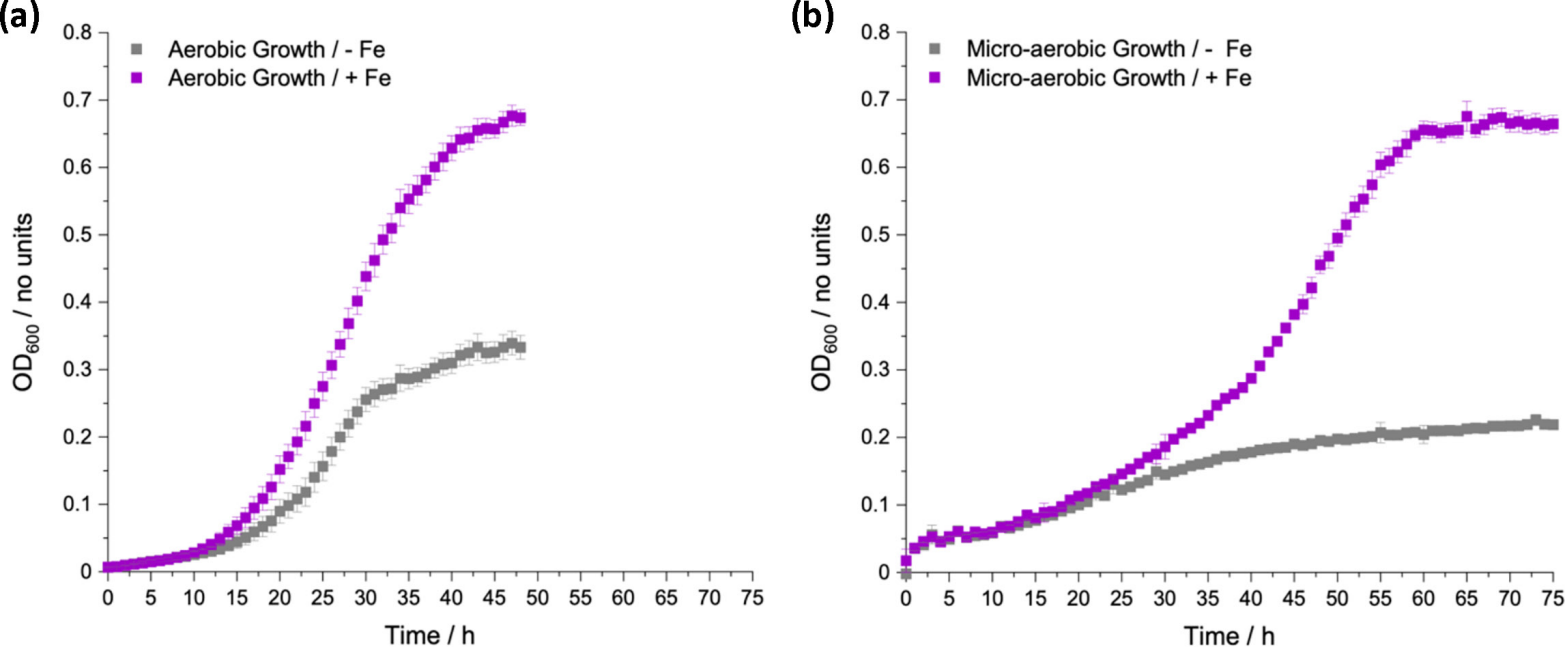
Aerobic (**a**) and microaerobic (2 % O_2_) (**b**) growth of *E. coli* K-12 (BW25113) in zero-iron media under iron-depleted (no added FeCl_3_, grey) and iron-replete (100 µM FeCl_3_, purple) conditions, at 37 °C for 48 and 75 h respectively.

Using the zero-iron media we investigated whether iron-limited growth under microaerobic conditions would be attenuated further compared to aerobic growth (Fig. 2). To this end, the same preculture was used as for aerobic growth but for microaerobic assays the plates were prepared in an anaerobic chamber using degassed zero-iron media, and their subsequent growth was measured under a microaerobic atmosphere in the absence and presence of added iron. The chosen oxygen percentage for these growth experiments was 2 % as, apart from the duodenum region of the colon, most other intestinal regions possess oxygen percentages of 2 % or lower [38].

In the presence of added iron, growth in either condition resulted in similar final growth yields, where microaerobic growth was much slower than aerobic growth, as expected due to less efficient respiration [39]. Interestingly, with the non-iron supplemented zero-iron media, growth in microaerobic conditions was also slower than in aerobic conditions but reached a final OD_600_ of around 0.2 (Fig. 2b) compared to over 0.3 for aerobic conditions (Fig. 2a), suggesting a limit on the overall growth yield consistent with an increased demand for iron-containing proteins utilized in microaerobic compared to anaerobic metabolism [40]. Therefore, another simple way to enhance the iron-limiting phenotype of zero-iron media would be to grow *E. coli* under microaerobic conditions.

### Use of exogeneous siderophores to induce extreme iron limitation

The most common hydroxymate-based siderophore used by *E. coli* is DFO [7,41, 42]. The use of DFO by *E. coli* is known, but the identities of the receptors required for its uptake across the outer membrane are still under discussion. Research by Hantke *et al.* reported iron taken up via DFO is present at low levels, compared to that of coprogen- or ferrichrome-mediated uptake, and therefore observing a growth enhancement on addition of DFO is more challenging [43,45]. Furthermore, they state this occurs by the TonB-dependent receptor FhuE [43,46]. An additional receptor called FoxB was reported by Nelson *et al.* [47,48].

**Fig. 3.**
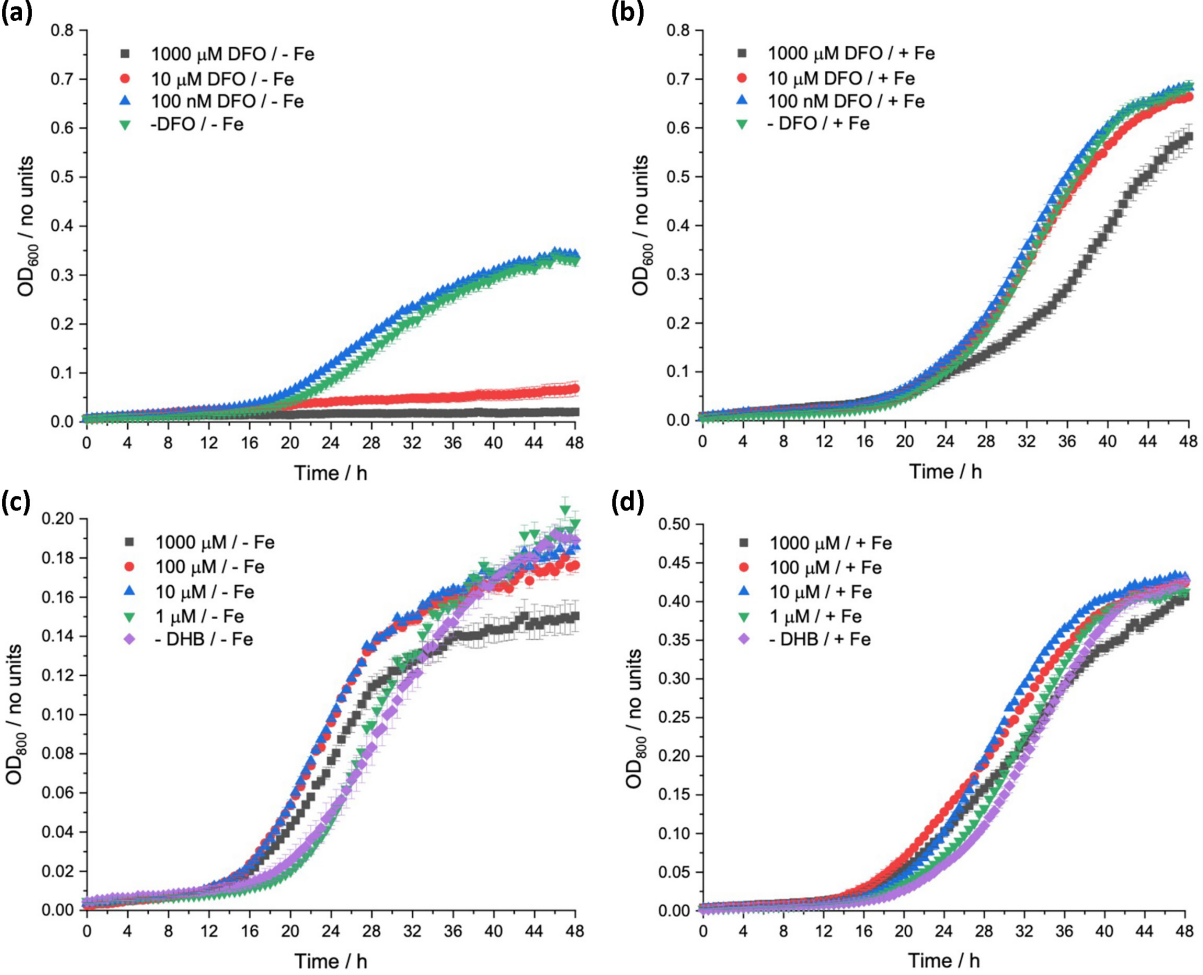
Growth of *E. coli* K-12 (BW25113), in zero-iron media, over 48 h at 37 °C, with varied concentration of DFO (**a, b**) and DHB (**c, d**) in iron-depleted (no added iron) and iron-replete conditions (100 µM FeCl_3_), respectively.

The effect of DFO addition for aerobic *E. coli* growth was measured using iron-depleted and iron-replete zero-iron media (Fig. 3a, b, respectively). Under iron-depleted conditions DFO completely inhibits growth at concentrations above 0.1 µM. Under iron-replete conditions, this inhibition is completely removed for concentrations <10 µM, suggesting the main mode of action is associated with its ability to compete for iron binding. As DFO binds strongly to iron, it competes with native, bacteria-released siderophores, and the iron uptake mediated by native siderophores will decrease accordingly, leading to a decrease in bacterial growth. This hypothesis is supported by studies by Muller *et al.*, who stated the biosynthesis of unrelated bacterial siderophores is stimulated by the presence of DFO in solution, suggesting DFO-mediated uptake alone is insufficient to maintain optimum iron levels in bacteria [49,50]. Therefore, it can be concluded that DFO is not an effective siderophore for these bacteria and can be used to impose iron limitation. Moreover, these studies serve as a useful reminder that siderophore compatibility with target pathogens should be investigated for the development of Trojan Horse antibacterials.

Hancock *et al*. [51] investigated the DHB-mediated uptake of iron in *E. coli*. They concluded that the uptake of DHB–iron complexes occurred via diffusion across the outer membrane, followed by capture of the iron by enterobactin or a 2,3-dihydroxybenzoylserine (DBS) dimer or trimer in the periplasm where translocation into the cytoplasm occurred [51]. DBS is a hydrolysis product of enterobactin and used as a siderophore by *E. coli* mainly via receptors Fiu and FepA [52,53]. These results were reinforced by growth response assays by Winkelmann *et al.* [54] and by aerobic growth experiments carried out in this work, with varied DHB concentration in iron-depleted and iron-replete zero-iron media (Fig. 3c and d respectively) [51].

OD_800_ was measured as iron–catechol complexes absorb at 600 nm. Under iron-depleted conditions, addition of DHB at 1 µM had no effect on growth, while concentrations of 10 and 100 µM gave a reduced lag phase and increased growth rate compared to no addition (Fig. 3c). This effect was reversed by the addition of 1 mM DHB, which reduced the growth rate slightly and decreased the final growth yield, an effect attributed to excessive iron binding, impeding iron complex formation with the siderophores released by the bacteria. These effects were observed although diminished in the iron-replete medium as cultures grew more similarly, with no differences in the final growth yield (Fig. 3d). Expectedly, with its known involvement in iron-uptake pathways for *E. coli* and relatively weaker binding to iron compared to DFO, DHB was found to be less suitable for imposing enhanced iron limitation.

### Summary and conclusions

This study was initiated by problems imposing iron limitation for *E. coli* using a commercial preparation of the M9 minimal salts medium, widely used in physiological studies of *E. coli* growth. From a combination of our own analysis of iron contamination by inductively coupled plasma MS [32], previous conclusions made by Neidhardt *et al*. [33] and a very recent study by Soma *et al*. [34] that showed highly variable levels of contaminants in commercial M9 salts, we conclude that M9 should be avoided as a base medium for iron-limitation studies and would recommend the MOPS-based, zero-iron medium we have used here.

We were able to improve the strength of the limitation phenotype further by altering the physiological conditions of growth, presumably by increasing demand for iron-containing proteins. Another strategy was also tested by the addition of suitable exogeneous siderophores, which in one case completely inhibited bacterial growth, perhaps mimicking the situation inside a host organism. The addition of DHB at concentrations of 10 and 100 µM reduced lag phases, indicating enhanced iron uptake, but at 1 mM, perturbed growth was observed that was attributed to competitive iron binding. A similar mode of action was observed for DFO but with more pronounced potencies due to the siderophore’s stronger iron binding. Interestingly, in iron-replete conditions, this toxicity was reversed, indicating its mode of action was indeed the withholding of iron from the bacteria rather than other effects on the cells. Thus, one might simply conclude DHB is insufficient to impose iron limitation at the tested concentrations for this *E. coli* strain whereas DFO possesses potent iron withholding abilities associated with its higher iron affinity, which combined with its poor uptake means it can strongly inhibit growth when added exogenously. Although outside the scope of this study, future work could include the growth of siderophore transporter deletion mutants in the presence of DHB and DFO, to confirm and identify their means of internalization.

The findings reported here highlight the importance of media optimization prior to their use and have contributed to our wider understanding of bacterial iron homeostasis. This work provides a foundation for further research into iron-dependent bacterial processes, and we strongly encourage future studies in the area of bacterial growth for mimicking host conditions to utilize zero-iron media, such as the one used in this work.

## Abbreviations

DBS: 2,3-dihydroxybenzoylserine
DFO: desferrioxamine
DHB: 2,3-dihydroxybenzoic acid
DNA: deoxyribonucleic acid
*E. coli*: *Escherichia coli*
LB: Lysogeny broth
MHII: Müller-Hinton broth II
MOPS: 3-(N-morpholino)propanesulfonic acid
OD: optical density
*S. aureus*: *Staphylococcus aureus*
TMS: tris-minimal succinate
TSB: tryptic soy broth
