# Corrigendum: Enhancement of growth media for extreme iron limitation in Escherichia coli

**DOI:** 10.1099/acmi.0.000887

**Published:** 2024-08-01

**Authors:** James W. Southwell, Keith S. Wilson, Gavin H. Thomas, Anne-Kathrin Duhme-Klair

**Affiliations:** 1 Department of Chemistry, University of York, Heslington, York, YO10 5DD, UK; 2 York Structural Biology Laboratory, University of York, Heslington, York, YO10 5DD, UK; 3 Department of Biology, University of York, Wentworth Way, York, YO10 5DD, UK

An external reader and the author of the article ‘A hybrid in silico/in-cell controller for microbial bioprocesses with process-model mismatch’ contacted the Editorial Office suggesting possible mistake in the reference 34 in the published article. Authors confirm that the wrong article was cited as reference 34 by mistake.

The corrected reference 34 is:

**34. SomaY**, **TominagaS**, **TokitoK**, **ImasoY**, **NakaKet al**. Trace impurities in sodium phosphate influences the physiological activity of *Escherichia coli* in M9 minimal medium. *Sci Rep 2023*; 13: https://doi.org/10.1038/s41598-023-44526-4

Previously, the reference 34 appeared as shown below:

**34. OhkuboT**, **Soma Y**, **Sakumura Y**, **Hanai T**, **Kunida K**. A hybrid in silico/in-cell controller for microbial bioprocesses with process-model mismatch. *Sci Rep* 2023; 13 : 13608

